# The transcription factor NR4A1 is essential for the development of a novel macrophage subset in the thymus

**DOI:** 10.1038/srep10055

**Published:** 2015-06-19

**Authors:** Robert Tacke, Ingo Hilgendorf, Hannah Garner, Claire Waterborg, Kiwon Park, Heba Nowyhed, Richard N. Hanna, Runpei Wu, Filip K. Swirski, Frederic Geissmann, Catherine C. Hedrick

**Affiliations:** 1 Division of Inflammation Biology, La Jolla Institute for Allergy and Immunology, La Jolla, California, USA; 2 Centre for Molecular and Cellular Biology of Inflammation, King’s College London, London, UK; 3 Center for Systems Biology, Massachusetts General Hospital and Harvard Medical School, Boston, Massachusetts, USA

## Abstract

Tissue macrophages function to maintain homeostasis and regulate immune responses. While tissue macrophages derive from one of a small number of progenitor programs, the transcriptional requirements for site-specific macrophage subset development are more complex. We have identified a new tissue macrophage subset in the thymus and have discovered that its development is dependent on transcription factor NR4A1. Functionally, we find that NR4A1-dependent macrophages are critically important for clearance of apoptotic thymocytes. These macrophages are largely reduced or absent in mice lacking NR4A1, and *Nr4a1*-deficient mice have impaired thymocyte engulfment and clearance. Thus, NR4A1 functions as a master transcription factor for the development of this novel thymus-specific macrophage subset.

Macrophages are a heterogeneous population of phagocytic cells that are present in all tissues throughout the body^1^. At steady state, macrophages function in the maintenance of tissue homeostasis through the release of cytokines and growth factors, and through engulfment of apoptotic cells^2^. In times of stress (be it physical trauma or infection), macrophages clear invading pathogens and cellular debris, recruit and activate surrounding immune cells, and mediate wound repair^3^. Importantly, tissue-specific macrophages have also developed site-specific functions in order to best support the tissue that they inhabit^4^. Examples of this phenomenon are observed in the spleen where tissue-resident red pulp macrophages function to engulf aging red blood cells and recycle heme-associated iron^1^, and also in the bone where macrophages known as osteoclasts function as bone resorbing cells to maintain skeletal health^5^,^6^. Tissue-specific macrophages not only vary in function, but also have different transcriptional requirements. For example, *Spic* is required for the development of splenic red pulp macrophages but is dispensable for the development of other tissue macrophages^7^. Likewise, *Gata6* expression is essential for tissue-specific development and function of peritoneal macrophages^4^,^8^.

We have previously reported that the transcription factor NR4A1 controls the development of the non-classical, patrolling Ly6C^−^ subset of monocytes. However, a role for NR4A1 in tissue macrophage development has not been determined. In this study, we assessed the capacity of NR4A1 to regulate tissue-resident macrophage development by examining macrophage frequencies in a variety of tissues in *Nr4a1*-deficient mice. We discovered a requirement for *Nr4a1* in the development of a novel subset of macrophages present in the thymus. While macrophage populations present in the thymus have not been thoroughly characterized, it has long been appreciated that thymic macrophages are critical to the engulfment of apoptotic thymocytes; cells that are constantly being generated during both positive and negative selection in lymphocyte development^9^. In this report we show that the population of NR4A1-dependent thymic macrophages actively engulfs apoptotic thymocytes. Further, we show that this NR4A1-dependent subset of thymic macrophages likely derives from bone marrow-progenitors, but does not depend on short-lived circulating monocytes for cell renewal.

## Methods

### Mice

C57BL/6J wild-type mice (000664), *Nr4a1*^−/−^ (006187), *Ccr2*^rfp/rfp^ (017586), *Cx3cr1*^gfp/gfp^ (005582), *Csf1r*^*eGFP*^ (018549), *Csf1*^op^ (000231), and B6.SJL-*Ptprc*^*a*^ *Pepc*^*b*^/BoyJ mice (002014) were obtained from The Jackson Laboratory. CD45.1.2 mice were generated by crossing C57BL/6J (CD45.2) mice to B6.SJL-*Ptprc*^*a*^*Pepc*^*b*^/BoyJ (CD45.1) mice. Generation of *Flt3*^cre/wt^;Rosa26^LSL-eYFP/wt^ mice has been described previously^10^. *Nr4a1*^flox^ mice were a gift from Professor Pierre Chambon, Institut de Genetique et de Biologie Moleculaire et Cellulaire, Illkirch-Graffenstaden, France. *Nr4a1*^fl/fl^ mice were crossed to *Csf1r*^cre/wt^ (Jackson, 021024) mice and the resulting *Nr4a1*^fl/fl^*;Csf1*^cre/wt^ mice were analyzed with *Nr4a1*^wt/wt^*;Csf1*^cre/wt^ littermates used as controls. *Nr4a1*^eGFP/cre^ (Jackson, 016617) mice were crossed to Rosa26^tdTomato^ (Jackson, 007905) mice and the resulting *Nr4a1*^eGFP/cre+^;Rosa26^tdTomato+^ mice were analyzed using cre-negative littermates as controls. All experiments followed guidelines of the La Jolla Institute for Allergy and Immunology Animal Care and Use Committee, and approval for the use of rodents was obtained from the La Jolla Institute for Allergy and Immunology according to criteria outlined in the Guide for the Care and Use of Laboratory Animals from the National Institutes of Health. Mice were euthanized by CO_2_ asphyxiation and cervical dislocation.

### Tissue Processing

Blood was drawn from mice via cardiac puncture with an EDTA-coated syringe. Thymus and spleens were excised and pushed through a 70-μm strainer, and bone marrow cells from both femurs and tibias were collected by centrifugation. Brains were harvested from mice perfused with 10 mL Dulbecco’s PBS (Gibco), minced into small pieces, and digested in RPMI 1640 (Gibco) with 300 μg/mL Liberase, and 100 U/mL DNase-I for 30 min at 37 °C. The tissue was then passed through a 70-μm strainer and mononuclear cells were enriched by 30% Percoll-gradient centrifugation. All samples were collected in Dulbecco’s PBS (Gibco) and were stored on ice during staining and analysis. Red blood cells were lysed in RBC Lysis Buffer according to the manufacturer’s protocol (BioLegend).

### Detection of anti-nuclear antibodies

Serum was collected from wild type and Nr4a1^−/−^ animals following blood collection by cardiac puncture at the indicated times. Serum was isolated via centrifugation. To measure anti-nuclear antibodies, poly-L-lysine-coated EIA plates were first coated with genomic DNA. After the plates were blocked with 5% FBS in PBS, diluted mouse serum was added to each well and incubated at 4°C for 16 hrs. Horseradish peroxidase-conjugated anti-mouse IgG was used to detect DNA recognizing IgG. Peroxidase-mediated fluorescence was initiated using TMB substrate. Absorbance at 450 nm was measured by SpectraMax M5 (Molecular Device) ELISA reader. Data was analyzed by Softmax Pro3.6 software.

### Flow Cytometry

5 × 10^6^ cells were resuspended in 100 μl staining buffer (1% BSA, 0.1% sodium azide in PBS). Cells were blocked with Fcγ receptor for 15 min and stained for surface antigens with flow cytometry Abs for 30 min at 4 °C. A fixable LIVE/DEAD stain (Invitrogen) was used to measure viability, and FSC/SSC parameters were used to exclude doublets from analysis. For intracellular staining, cells were treated using the Foxp3 Staining Kit (BD) according to the manufacture’s instructions. Cellular fluorescence was determined using a LSRII, or a FACSCalibur flow cytometer (BD Biosciences), and data analyses were performed with FlowJo software (Tree Star). The antibodies used in this study are shown in Supplementary Table 1.

### Fluorescent Activated Cell Sorting (FACS)

Prior to sorting, thymus cells were enriched for non-T cells via negative selection using the StemSep^tm^ Anti-APC selection kit (StemCell Technologies) in conjunction with APC-conjugated anti-CD90 (eBioscience) according to manufacturer’s instructions. Cells were then stained for surface antigens as described above and sorted using a FACSAria cell sorter (BD Biosciences). Approximately 5 × 10^4^ events were collected, and used in qPCR, and *in vitro* experiments. An aliquot of thymic CD11b^−^F4/80^+^ cells were cytospun onto a microscope slide, stained with Hema3 (Fisher Scientific), and analyzed by miscroscopy.

### Quantitative Real-Time PCR (qRT-PCR)

Total cellular RNA was collected from whole thymic tissue or from FACS isolated thymic macrophage populations using a Qiagen RNeasy Plus Micro Kit following the manufacturer’s protocol. RNA purity and quantity was measured using a nanodrop spectrophotometer (Thermo Scientific). Approximately 500 nanograms of RNA was used to synthesize cDNA using an iscript cDNA synthesis kit (Bio-Rad). Total cDNA was diluted 1:15 in H_2_O, and 9 μl were used for each real-time condition using a Bio-Rad MyIQ single-color real-time PCR detection system. Taqman primers and TaqMan Gene Expression Mastermix was used for the genes glyceraldehydes-3-phosphate dehydrogenase (GAPDH), Mertk, TYRO3, IL6, IL10, IL23a, and TNF (Supplementary Table 2). For the genes AXL, CD68, milk fat globule epidermal growth factor (EGF) factor 8 (MFGE8) and stabilin-2 (STAB2), SYBR Green primers (Supplementary Table 3) and SYBR Green Gene Expression Mastermix (Qiagen) were used. Data were analyzed and presented on the basis of the relative expression method. The formula for this calculation is as follows: relative expression = 2^−(SΔ *Ct* − CΔC *t*)^ where Δ*C*_*t*_ is the difference in the threshold cycle between the gene of interest and the housekeeping gene (GAPDH), S are the CD11b^−^F4/80^+^ cells or *Nr4A1*^−/−^ tissue, and C are the CD11b^+^F4/80^+^ cells or wild type tissue.

### *In vitro* engulfment and phagocytosis assays

Sorted CD11b^+^F4/80^+^ and CD11b^−^F4/80^+^ macrophages were incubated with apoptotic thymocytes for 1hr.. Uptake of labeled-thymocytes by the sorted macrophages was measured by flow cytometry. For the induction of apoptosis, thymocytes were incubated with 50 μM Dexamethasone (Calbiochem Inc.) at 37 °C for 4 hours. The thymocytes were then washed and stained with pHrodo-succinimidyl ester (SE) (Molecular Probes), a pH-sensitive fluorescent dye used to assess engulfment due to the increase in the intensity of pHrodo light emission following the pH change once engulfed by a macrophage^11^. PHrodo-labeled thymocytes were then co-cultured with the sorted macrophages at a ratio of 10:1 (labeled thymocytes to sorted phagocytes). Forward and side-scatter parameters, as well as CD11b and F4/80 staining, were used to distinguish free unbound targets from phagocytes.

### *In vivo* induction of thymocyte apoptosis

Wild type and *Nr4a1*-deficient mice were intraperitoneally injected with 200 μl of PBS containing 250 μg dexamethasone dissolved in ethanol. 18 hours after injection, thymi were harvested and processed as described above, and stained for Annexin V and 7AAD using the PE Annexin V Apoptotis Kit I (BD Biosciences). Thymi were also embedded in OCT and then frozen on dry ice.

### Histology

Embedded thymi were sectioned (10 μm) using a cryostat. The slides were subsequently fixed with 4% paraformaldehyde for 20 min. Sections stained for cleaved caspase-3 were permeablized in a 0.1% citrate, 0.1% Triton X solution for 10 min. The slides were then washed 3 times in PBS, blocked in 5% donkey serum with 0.1% Triton X in PBS for 60 min, and then incubated with a 1:500 dilution of rabbit anti-mouse cleaved caspase-3 (Asp175) antibody from Cell Signaling (#9661) or with a 1:50 dilution of PE-conjugated anti-TIM4 (RMT4-54, eBioscience) diluted in PBS with 0.2% Triton X and 1% BSA overnight at 4 °C. The following day, Caspase-3 stained slides were then washed 3 times in PBS and incubated for 1 hour at room temperature with a donkey anti-rabbit Alexa488 fluorescently conjugated secondary antibody (Invitrogen). The slides were then stained with DAPI, washed 3 more times in PBS, and mounted with ProlongGold (Invitrogen). Images were acquired using an Olympus FV10i confocal microscope and analyzed using Imaris software (Bitplane).

### Bone marrow transplantation studies

For bone marrow transplants, recipient mice (CD45.1) were irradiated in two doses of 550 rads each (for a total of 1,100 rads) 4 h apart. Bone marrow cells from both femurs and tibias of donor mice (wild-type CD45.2 or *Nr4a1*^−/−^) were collected under sterile conditions. Approximately 5 × 10^6^ nucleated bone marrow cells were obtained from each donor mouse. Bones were centrifuged for the collection of marrow, then cells were washed and resuspended in Dulbecco’s PBS for injection. Approximately 5 × 10^6^ unfractionated bone marrow cells in 200 μl media were delivered retro-orbitally into each recipient mouse. Recipient mice were housed in a barrier facility under pathogen-free conditions before and after bone marrow transplantation. After bone marrow transplantation, mice were provided autoclaved acidified water with antibiotics (trimethoprim-sulfamethoxazole) and were fed autoclaved food. Mice were used for experiments after 7 or 8 weeks of bone marrow reconstitution as indicated. In mixed bone marrow chimera studies 2.5 × 10^6^ cells from *Nr4a1*^−/−^ mice (CD45.2) and 2.5 × 10^6^ cells from B6.CD45.1 mice were mixed at a ratio of 1:1 for reconstitution of recipients (wild-type, CD45.1) as described above.

### Parabiosis

The procedure was performed as previously described^12^. Briefly, after shaving the corresponding lateral aspects of each mouse, matching skin incisions were made from behind the ear to the tail of each mouse, and the subcutaneous fascia was bluntly dissected to create about 0.5 cm of free skin. The scapulas were sutured using a mono-nylon 5.0 (Ethicon, Albuquerque, NM), and the dorsal and ventral skins were approximated by continuous suture. Mice were joined for 4 weeks, and thymus and spleen were then collected and analyzed as described above.

### Statistical analysis

Data for all experiments were analyzed using Prism software (GraphPad, San Diego, CA). Unpaired *t* tests were used to compare experimental groups. All experiments were repeated at least 3 times with 3 or more individuals per group. Data are graphically represented as mean ± SEM. A *P* value <0.05 was considered significant.

## Results

### Identification of a new subset of thymic macrophages and the dependence on NR4A1 for their development

**Figure 1:**
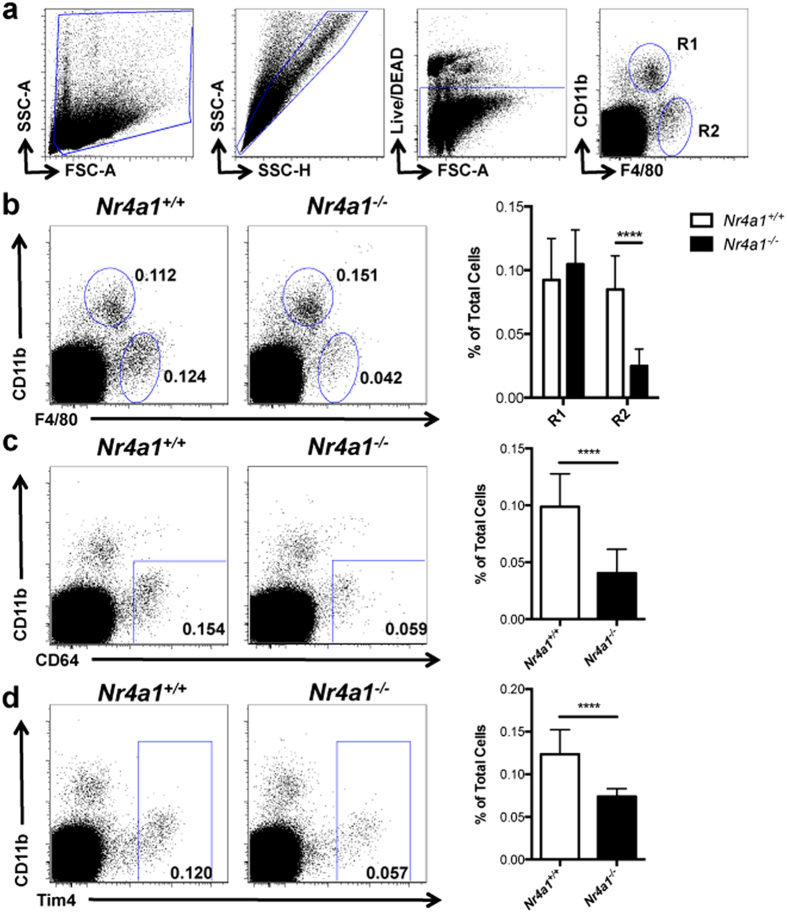
Identification of a new NR4A1-dependent macrophage subset in the thymus. (**a**) Gating strategy for F4/80^+^ cells in the thymus. (**b-d**) *Nr4a1*^+/+^ and *Nr4a1*^−/−^ cells from the thymus were stained with antibodies to CD11b, F4/80, CD64, and Tim4 and analyzed by flow cytometry. Frequency calculated as a percent of the total, live, singlet cells. Data are representative of at least three independent experiments (n = 14 mice). *** P < 0.001

**Figure 2:**
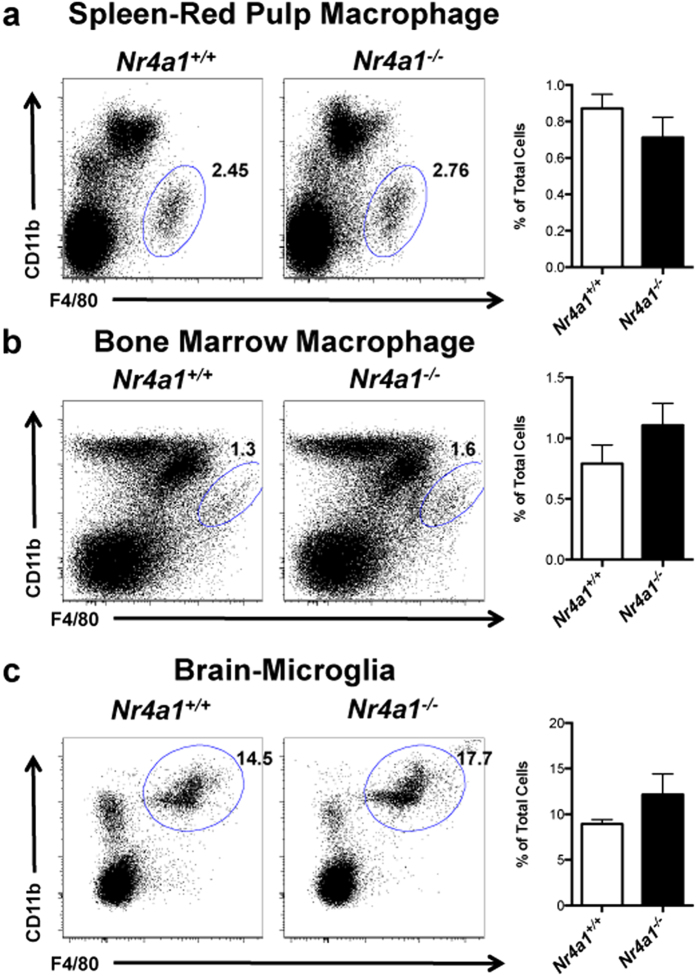
Site specific regulation of tissue macrophages by NR4A1. *Nr4a1*^+/+^ and *Nr4a1*^−/−^ cells from the indicated tissues were stained with antibodies to F4/80 and CD11b and analyzed by flow cytometry. Frequency calculated as a percent of the total, live, singlet cells in the corresponding tissue. Data are representative of at least three independent experiments (n = 9 mice).

**Figure 3:**
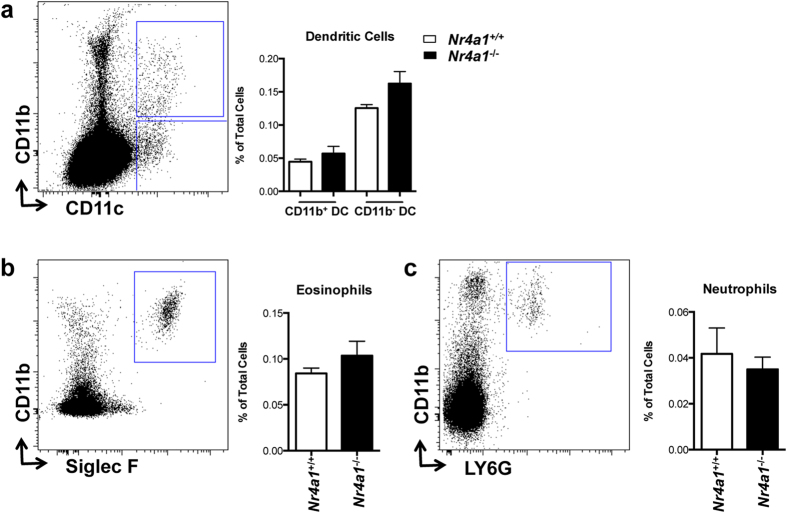
Other myeloid cell populations in the thymus are unaffected in *Nr4a1*^−/−^ mice. Thymus cells from *Nr4a1*^+/+^ and *Nr4a1*^−/−^ mice were stained with antibodies to CD11b, CD11c, Siglec F, and Ly6G and analyzed by flow cytometry. (**a**) Dendritic cells were identified as CD11b^+^CD11c^+^ or CD11b^−^CD11c^+^ cells; (**b**) eosinophils as CD11b^+^Siglec F^+^ cells; (**c**) neutrophils as CD11b^+^Ly6G^+^ cells. Frequency calculated as a percent of the total, live, singlet cells in the thymus. Data are representative of three independent experiments (n = 9 mice).

To determine if NR4A1 functions in tissue macrophage development, we harvested brain, spleen, bone marrow, and thymus from *Nr4a1*^−/−^ and wild type mice and examined each for the presence of tissue macrophages, identified as F4/80^hi^ cells with varying levels of CD11b expression among the different tissues^1^,^13^,^14^. We found that the frequency of CD11b^−^F4/80^+^ cells in the thymus was reduced in *Nr4a1*-deficient mice by over 60% (Fig. 1b**, R2**), whereas CD11b^+^F4/80^+^(**R1**) cells were unaltered. In the thymus, we utilized macrophage markers CD64 and Tim4 as additional means to assess the presence of thymus-resident macrophages, and found a similar reduction of CD11b^−^CD64^+^, and CD11b^−^Tim4^+^ cells in *Nr4a1*-deficient mice (Fig 1b,c). Tissue macrophage development in brain, spleen, and bone marrow was also unaffected indicating that *Nr4a1*-deficient mice do not display a global macrophage deficiency (Fig. 2). Examination of other myeloid cell populations in thymus of *Nr4a1*-deficient mice revealed no changes in the frequency of neutrophils, eosinophils, cDCs (CD11b^+^CD11c^+^), or CD11b^−^CD11c^+^ DCs (Fig. 3). Given the substantial defect within the thymic CD11b^−^F4/80^+^ subset in *Nr4a1*^−/−^ mice, we chose to focus our investigation on these cells.

**Figure 4:**
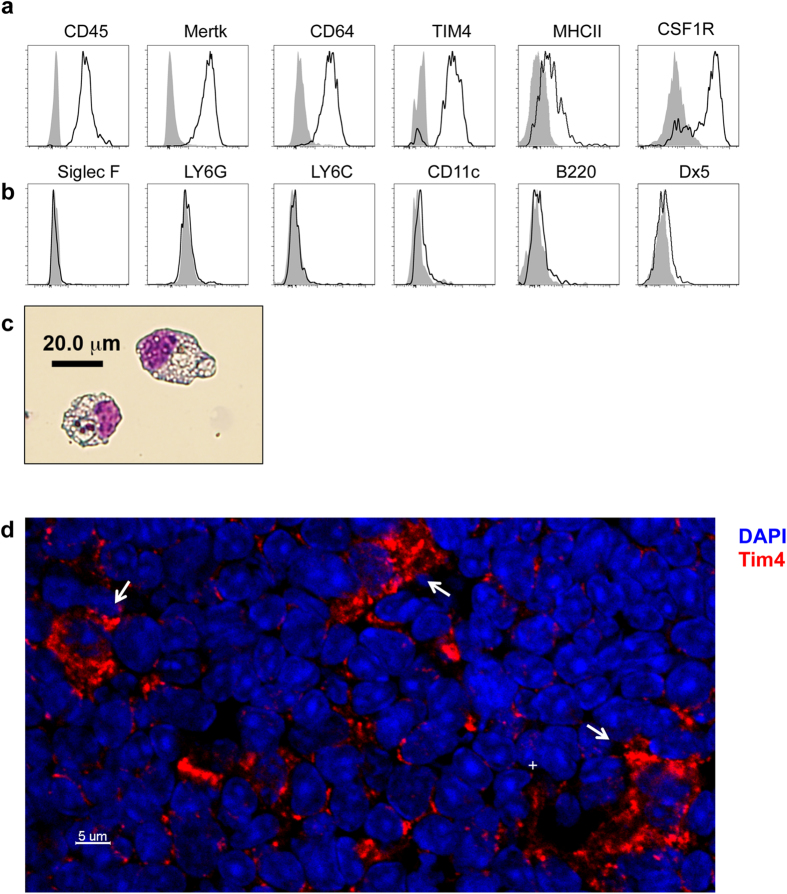
Thymic CD11b^−>^F4/80^+^ cells have a macrophage signature. (**a**,**b**) Thymus cells from C57BL/6 mice were stained with the indicated antibodies and analyzed by flow cytometry. Plots were first gated on live CD11b^−^F4/80^+^ cells. Shaded histograms represent isotype controls. In the case of CSF1R expression, *CsF1r*-GFP^+^ mice were used, and the shaded histogram is derived from a *Csf1r*-GFP^−^ mouse. (**c**) Hema3 staining of thymic CD11b^−^F4/80^+^ cells sorted and cytospun from C57BL/6 mice. (**d**) Thymic sections from C57BL/6 mice were stained with DAPI (blue) to mark the nucleus and antibodies to Tim4 (red). Arrows indicate Tim4^+^ macrophages. All data representative of three independent experiments.

While the presence of macrophages in the thymus has long been appreciated, thymic macrophage subsets have not been well characterized either phenotypically or functionally. We examined cell surface molecules expressed by this thymic subset of CD11b^−^F4/80^+^ macrophages by flow cytometry, and found that they express CD45 and mononuclear phagocyte-associated (MPS; monocyte, macrophage, and dendritic cells) growth factor receptor CSF1R (Fig 4a). Furthermore, much like a wide array of MPS cells^5^, thymic CD11b^−^F4/80^+^ macrophages are also CSF-1 dependent as *Csf-1*-deficient mice (*op/op*) display defects in thymic CD11b^−^F4/80^+^ cell development (>60% reduction) that closely mirror that of *Nr4a1*-deficient mice (Supplemental Fig. 1). Thymic CD11b^−^F4/80^+^ cells also express the efferocytosis receptors Mertk and Tim4, as well as Fc IgG receptor CD64, and intermediate levels of MHCII (Fig. 4a). Dual expression of CD64 and Mertk is associated with mature tissue resident macrophages in spleen, brain, lung, and peritoneum^14^. Tissue resident macrophages in the peritoneum also express high levels of Tim4^15^. Therefore, co-expression of Mertk, CD64, and Tim4 further supports the hypothesis that these CD11b^−^F4/80^+^ cells are part of the tissue resident macrophage population in the thymus. Importantly, thymic CD11b^−^F4/80^+^ cells also fail to express markers used to characterize a wide variety of leukocytes including inflammatory monocytes (Ly6C), DCs (CD11c), eosinophils (Siglec F), granulocytes (Ly6G), NK cells (Dx5), and B cells (B220) (Fig. 4b). Thymic CD11b^−^F4/80^+^ cells possess a macrophage-like morphology (Fig. 4c) that is also observed *in situ* via histological analysis of frozen thymic sections using confocal microscopy (Fig. 4d). Tim4 was used as a marker for thymic CD11b^−^F4/80^+^ cells during the histological analysis due to the exclusive expression within this population in the thymus (Supplemental Fig. 2).

### NR4A1-dependent thymic CD11b^−^F4/80^+^ cells are derived from bone marrow cells

**Figure 5:**
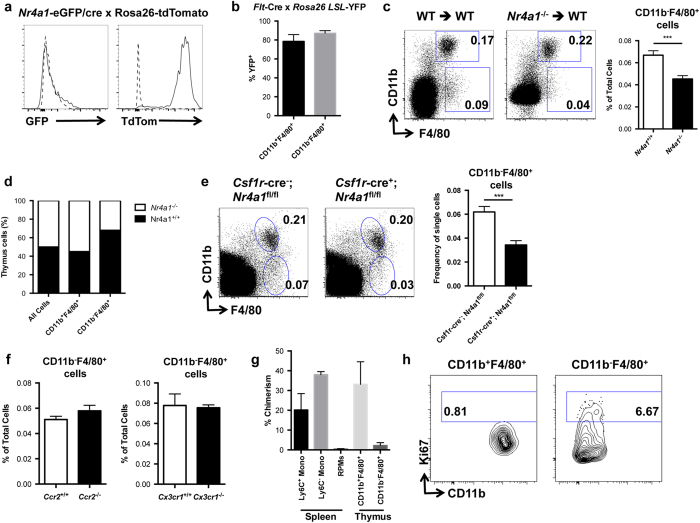
NR4A1-dependent thymic CD11b^−^F4/80^+^ macrophages derive from the bone marrow. (**a**) Thymus cells from *Nr4a1*-eGFP/cre × Rosa26-tdTomato mice were gated on the CD11b^−^F4/80^+^ population and eGFP and tdTomato expression was assessed by flow cytometry. (**b**) Thymus cells from *Rosa26-*YFP^+^ mice were gated on the CD11b^−^F4/80^+^ population and YFP expression was assessed by flow cytometry. (**c**) Bone marrow cells from *Nr4a1*^+*/*+^(CD45.2) or *Nr4a1*^−/−^(CD45.2) mice were transferred into irradiated CD45.1 hosts. Reconstitution of thymic F4/80^+^ cells was assessed by flow cytometry after 7 weeks. Data are representative of at least three independent experiments (n = 9 mice). *** P < 0.001 (**d**) *Csf1r*^cre−^;*Nr4a1*^flox/flox^ and *Csf1r*^cre+^;*Nr4a1*^flox/flox^ thymus cells were stained with antibodies to F4/80 and CD11b and analyzed by flow cytometry. (**e**) Bone marrow cells from *Nr4a1*^+*/*+^(CD45.1.2) and *Nr4a1*^−/−^(CD45.2) mice were transferred at a 1:1 ratio into irradiated CD45.1 hosts. Reconstitution of thymic CD11b^−^F4/80^+^ cells from each donor were assessed after 8 weeks. Data representative of four independent experiments (n = 22 mice). (**f**) *Ccr2*^−/−^ and *Cx3cr1*^−/−^ thymus cells were stained with antibodies to F4/80 and CD11b and analyzed by flow cytometry. Data representative of two independent experiments (n = 6). (**g**) Chimerism for spleen monocytes (mono), splenic red pulp macrophages (RPM), thymic CD11b^+^F4/80^+^, and thymic CD11b^−^F4/80^+^ cells in mice that had been joined in parabiosis for 4 weeks. (**h**) Ki67 expression in thymic CD11b^+^F4/80^+^ and CD11b^−^F4/80^+^ cells. Data representative of three independent experiments (n = 8 mice).

Due to the requirement of NR4A1 for the development of thymic CD11b^−^F4/80^+^ macrophages, we assessed NR4A1 expression in these cells. This was accomplished by examining *Nr4a1*^eGFP/cre^ *×* *Rosa26*^tdTomato^ mice. These mice express tdTomato in any cell that has expressed NR4A1 during its development, and eGFP in cells currently expressing NR4A1. Surprisingly, we found that thymic CD11b^−^F4/80^+^ cells do not express eGFP, but highly express tdTomato (Fig. 5a). These data show that while thymic CD11b^−^F4/80^+^ cells do not constitutively express NR4A1, it is transiently expressed during their development.

To examine the developmental lineage of thymic CD11b^−^F4/80^+^ cells, we utilized a fate mapping strategy involving *Flt3*^*cre*^ *×* *Rosa*^*LSL*−*YFP*^ mice. Growth factor receptor FLT3 is transiently expressed in pluripotent hematopoietic progenitors, and so any cell derived from the hematopoietic system in these mice will express yellow fluorescent protein (YFP)^10^. Analysis of *Flt3*^*cre*^ *×* *Rosa*^*LSL*−*YFP*^ mice revealed that recombination was high in CD11b^+^F4/80^+^ thymic macrophages (>75%) as well as in NR4A1-dependent CD11b^−^F4/80^+^ thymic macrophages (>85%), indicating that these subsets are likely derived from bone marrow hematopoietic progenitors^10^ (Fig. 5b).

The observed defect in the development of thymic CD11b^−^F4/80^+^ cells in *Nr4a1*-deficient mice could result from either a cell-intrinsic defect, or from the loss of a required extrinsic factor produced by other cells in the thymus. To address this issue we first examined whether the *Nr4a1*-deficient bone marrow could effectively reconstitute CD11b^−^F4/80^+^ thymic macrophages by transplanting wild type or *Nr4a1*-deficient bone marrow into irradiated congenic wild-type recipients to assess the reconstitution of the macrophage compartments in the thymus after 7 weeks (Fig. 5c). We found that *Nr4a1*-deficient bone marrow failed to reconstitute the CD11b^−^F4/80^+^ subset in the thymus as we observed a defect in thymic CD11b^−^F4/80^+^ cell frequency (~40% reduction relative to control) that closely mirrors that of global *Nr4a1*-deficient mice.

Next, we performed mixed bone marrow chimera studies by transplanting a 1:1 mixture of bone marrow cells from wild type CD45.1.2 and *Nr4a1*-deficient CD45.2 donors into wild type CD45.1 recipients (Fig. 5d). After reconstitution, we observed that cell numbers in the thymus were reconstituted in a 1:1 ratio of wild type to *Nr4a1*-deficient cells confirming that the donor bone marrow was injected at a 1:1 ratio. However, we found a 3:1 ratio of wild type to *Nr4a1*-deficient cells within the thymic CD11b^−^F4/80^+^ macrophage population, illustrating that *Nr4a1*-deficient bone marrow cells are significantly outcompeted by wild type bone marrow cells in a cell-intrinsic fashion, and indicating that the development of this thymic macrophage population requires NR4A1. In contrast, the CD11b^+^F4/80^+^ macrophage population was reconstituted equally (1:1) by wild type and *Nr4a1*-deficient marrow, indicating that NR4A1 was critically and selectively important for the development of only the CD11b^−^F4/80^+^ macrophage population in thymus. These findings were corroborated by NR4A1 conditional knockout studies using *Nr4a1*^*flox*^ *×* *Csf1r*^*cre*^ mice where NR4A1 is only deleted in cells expressing CSF1R. Analysis of *Nr4a1*^*flox*^ *×* *Csf1r*^*cre*^ mice shows a significant reduction in CD11b^−^F4/80^+^ macrophages in the thymus (Fig. 5e).

At steady state, a large proportion of macrophages in a range of tissues including the brain and liver derive from HSC-independent yolk sac progenitors^10^. However, steady state macrophages from the lamina propria derive from circulating CCR2^+^ monocytes^16^. To determine if thymic CD11b^−^F4/80^+^ cells are monocyte-derived, we analyzed blood monocyte and thymic macrophage populations in both *Ccr2* and *Cx3cr1*-deficient animals and found the expected reductions of Ly6C^+^ and Ly6C^−^ blood monocytes respectively^17^,^18^ (Supplemental Fig. 3). However, no concomitant defects in thymic CD11b^−^F4/80^+^ macrophage development were observed in either mouse model (Fig. 5f), suggesting that these cells are likely not derived from adult circulating Ly6C^+^ or Ly6C^−^ blood monocytes. These studies also further illustrate the critical dependence of these macrophages on NR4A1 for their development, as *Nr4a1*-deficient mice have a similar blood monocyte profile (greatly reduced Ly6C^−^ monocyte numbers) to that of *Cx3cr1*^−/−^ mice^19^,^20^. To further examine whether thymic CD11b^−^F4/80^+^ macrophages could be monocyte-derived, we assessed the chimerism of CD11b^−^F4/80^+^ thymic macrophages under parabiotic conditions. To do this, wild-type CD45.2 and CD45.1 mice were joined for four weeks, and chimerism of splenic monocytes, splenic red pulp macrophages (RPMs), thymic CD11b^+^F4/80^+^, and thymic CD11b^−^F4/80^+^ macrophages was determined. We found the expected 20-30% chimerism within the splenic monocyte pool in the parabionts, mirroring that of circulating monocytes^12^. We also found high chimerism among the thymic CD11b^+^F4/80^+^ population but very low chimerism among RPM (~0.5%), suggesting circulating monocyte dependence and independence, respectively. By comparison, the thymic CD11b^−^F4/80^+^ chimerism was very low (~2%), which argues against the concept that short lived, circulating, adult monocytes replenish this population (Fig. 5g). Lastly, we examined whether thymic CD11b^−^F4/80^+^ cells could locally proliferate by staining with antibodies to nuclear proliferation antigen Ki67. We found that over 6% of thymic CD11b^−^F4/80^+^ macrophages stained positively for Ki67 whereas substantially fewer (~0.8%) of circulation-derived CD11b^+^F4/80^+^ macrophages were Ki67-positive (Fig. 5h). Together these data show that while thymic CD11b^−^F4/80^+^ cells likely derive from the bone marrow, they are not derived from circulating short-lived monocytes at steady state. Further, this population of macrophages is proliferative, and thus is likely capable of self-renewal in thymic tissue.

### NR4A1-dependent thymic CD11b^–^F4/80^+^ cells function as efferocytes to maintain thymic homeostasis

**Figure 6:**
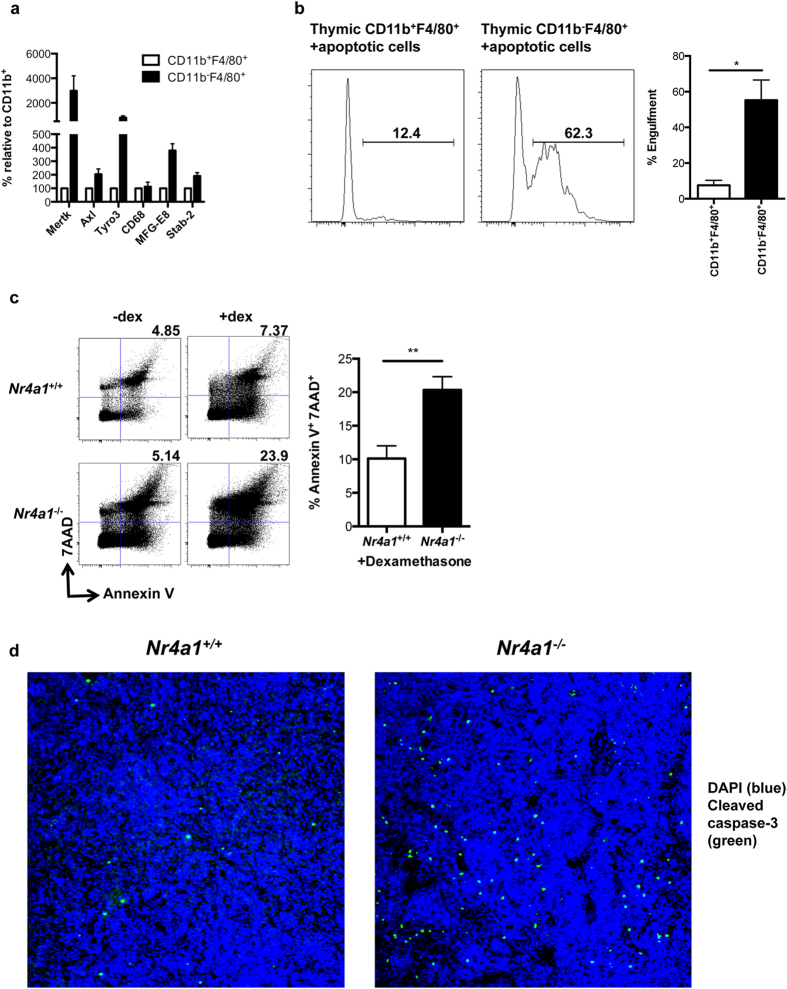
NR4A1-dependent thymic CD11b^−^F4/80^+^ macrophages function as efficient efferocytes. (**a**) Expression of efferocytosis genes in sorted thymic CD11b^+^F4/80^+^ and CD11b^−^ macrophages from C57BL/6 mice. Expression was detected by real time qPCR and calculated relative to CD11b^+^F4/80^+^ cells. Data from three independent experiments. (**b**) Efferocytosis of pHrodo-labeled apoptotic thymocytes by sorted thymic CD11b^+^F4/80^+^ macrophages and CD11b^−^F4/80^+^ macrophages. Efferocytosis was detected by flow cytometry. Data representative of three independent experiments. * *P* *<* *0.05* (**c**) 7AAD and Annexin V staining from *Nr4a1*^+*/*+^ and *Nr4a1*^−/−^ mice following 18 hr dexamethasone (dex) treatment. 8 animals per group. ** *P* < 0.01 (**d**) Thymic sections from *Nr4a1*^+/+^ and *Nr4a1*^−/−^ mice following 18hr dexamethasone treatment were stained with DAPI (blue) to mark the nucleus and antibodies to cleaved caspase-3 (green). Results representative of three mice per group.

Due to high turnover of thymocytes during positive and negative selection for T-lymphocyte development, one of the major functions of macrophages in the thymus is to clear apoptotic cells^9^. We therefore asked if this subset of CD11b^−^F4/80^+^ thymic macrophages was important in the efferocytosis of apoptotic cells in thymus. First, using qPCR, we found an enrichment of genes in wild-type CD11b^−^ thymic macrophages that are functionally associated with the engulfment of apoptotic cells (Fig. 6a). We found a several fold upregulation of a number of engulfment-associated genes including *Mertk*, *Axl*, *Tyro3*, and *Mfg-e8* in thymic CD11b^−^F4/80^+^ macrophages relative to CD11b^+^F4/80^+^ thymic macrophages in wild-type mice. Thus this population of thymic macrophages likely functions in mice to regulate efferocytosis.

Next, to examine the capacity of each macrophage population to engulf apoptotic thymocytes in the process of efferocytosis, sorted thymic CD11b^+^F4/80^+^ and CD11b^−^F4/80^+^ macrophages were incubated with pHrodo-labeled apoptotic thymocytes. After 1 hour of incubation, the sorted macrophage populations were assessed for their ability to engulf the apoptotic thymocytes by flow cytometry (Fig. 6b). We found that roughly 55% of cultured CD11b^−^F4/80^+^ macrophages engulfed apoptotic thymocyte targets, while only roughly 8% of CD11b^+^F4/80^+^ macrophages did. Therefore, these results indicate that thymic CD11b^−^F4/80^+^ macrophages function as highly efficient efferocytes, and are likely the major macrophage population in thymus that is responsible for clearing apoptotic thymocytes and maintaining thymic homeostasis.

Based upon these *in vitro* data, we wanted to see if Nr4a1-deficient mice were unable to efficiently clear apoptotic targets in the thymus *in vivo*. We injected wild type and *Nr4a1*-deficient mice with dexamethasone (Dex), a steroid that induces rapid apoptosis of thymocytes^21^. While wild-type animals showed only a modest increase in Annexin V^+^/7AAD^+^ cells 24 hr following Dex treatment, we observed a significant, two-fold accumulation of these late apoptotic/necrotic cells in *Nr4a1*^−/−^ mouse thymus *in vivo* (Fig. 6c). Further, as a control, we examined whether NR4A1 deficiency altered thymocyte sensitivity to Dex-induced apoptosis, and observed no difference in apoptotic cell induction between wild type and *Nr4a1*-deficient mice two hours following Dex injection (Supplemental Fig. 4). Apoptotic cell accumulation was also measured in thymus *in situ* following Dex injection of *Nr4a1*^−/−^ and wild type mice by assessing cleaved caspase-3 expression (Fig. 6d). We found greater accumulation of apoptotic caspase-3^+^ thymocytes in Dex-injected *Nr4a1*^−/−^ mice in thymus relative to wild type controls, again supporting the notion that this Nr4a1-dependent CD11b^−^F4/80^+^ macrophage subset plays an important role in clearance of apoptotic thymocytes during negative selection of lymphocytes in the thymus to aid in maintaining thymic homeostasis.

**Figure 7:**
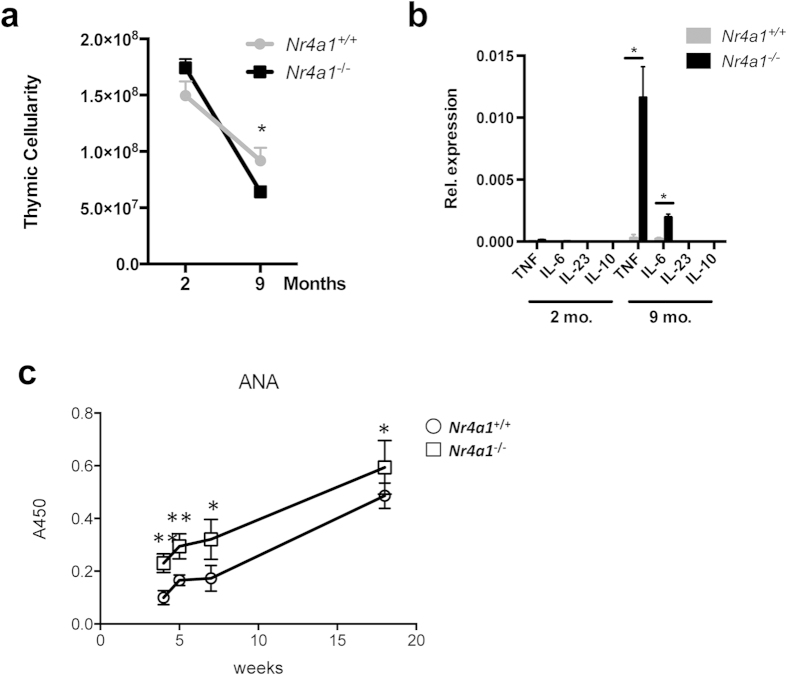
*Nr4a1*^−/−^ mice display accelerated thymic demise and increased auto antibody levels. (**a**) Cellularity of thymus from *Nr4a1*^+*/*+^ and *Nr4a1*^−/−^ mice at 8wk and 36wk. 8 mice per group. * *p* < *0.05* (**b**) Inflammatory gene expression in the thymus of *Nr4a1*^+*/*+^ and *Nr4a1*^−/−^ mice at 8wk and 36wk detected by real time qPCR. 6 mice per group. * *P < 0.05*. (**c**) Anti-DNA IgG (ANA) were measured from serum from mice aged from 4 to 18 weeks by ELISA. Anti-DNA antibody titer was significantly increased and peaked at 18 weeks in *Nr4a1*^−/−^. ‘*’, and ‘**’ indicated P < 0.05 and P < 0.005, respectively.

During the aging process the thymus begins to shrink in a process termed thymic involution^22^. As tissue macrophages are critical to maintenance of tissue homeostasis, we hypothesized that the loss of NR4A1-dependent thymic macrophages and the observed defective efferocytosis in these mice would alter homeostasis in the thymus and hasten thymic involution. To test this hypothesis, we examined thymic cellularity in both 8 week and 36 week-old wild type and *Nr4a1*-deficient mice, and found that while thymic cellularity was normal in *Nr4a1*-deficient mice at 8 weeks of age, at 36 weeks of age, the thymi of Nr4a1-deficient mice were significantly smaller (30% reduction in cellularity) than in wild type controls (Fig. 7a), indicating more rapid thymic demise in the *Nr4a1*^−/−^ mice. Because expression of proinflammatory cytokines in the thymus is associated with involution^23^, we measured the expression of the cytokines *Tnfa*, *Il6*, *Il23*, and *Il10* in thymus of wild type and *Nr4a1*-deficient animals at 8 weeks and 36 weeks of age (Fig. 7b). Consistent with our finding that *Nr4a1*-deficient mice have accelerated thymic involution at 36 weeks of age, we found elevated expression of both *Tnfa* and *Il6* in *Nr4a1*^−/−^ thymus at 36 weeks of age. Next, we assessed anti-nuclear antibody (ANA) production, a hallmark of a loss of self-tolerance and autoimmunity^24^. We found increased levels of anti-nuclear antibodies in the serum of *Nr4A1*^−/−^ mice, peaking at week 18, indicating that the observed dysregulation of thymic homeostasis may contribute to a loss of self-tolerance (Fig. 7c).

## Discussion

The transcription factor *Nr4a1* has been shown by our group and others to regulate both monocyte development and macrophage activation, but its capacity to regulate tissue macrophage development has not been investigated^19^,^25^,^26^. In this report, we identify a novel subset of CD11b^−^F4/80^+^ tissue macrophages in the thymus that selectively function to clear apoptotic thymocytes. Importantly, we show that NR4A1 is critical for the development of this thymic macrophage population, as these cells are drastically reduced in mice lacking *Nr4a1* expression in myeloid cells. It is likely that the remaining thymic CD11b^−^F4/80^+^ cells in *Nr4A1*^−/−^ mice represent an additional tissue macrophage population that is independent of NR4A1. Tissue macrophage development in the bone marrow, brain, and spleen in *Nr4a1*^−/−^ mice appeared normal (Fig. 2), indicating that *Nr4a1*-deficiency does not result in defects in global macrophage development. However, we do not rule out that NR4A1 may control the development of additional specialized macrophage subsets in tissues not examined in the current study.

NR4A1 functions as the master transcriptional regulator of the non-classical, patrolling subset of monocytes. Patrolling monocytes function by surveying the vasculature for both tissue damage and infection, however whether these cells are terminally differentiated or whether they extravasate into tissue and contribute to macrophage populations is currently unclear^27^. We hypothesized that any developmental defect observed within tissue macrophage populations in *Nr4a1*-deficient mice would likely be due to the absence of patrolling monocytes. However, analysis of *Cx3cr1*-deficient mice, which function as an alternative model of patrolling monocyte deficiency, showed no defect in CD11b^−^F4/80^+^ thymic macrophage subset development (Fig. 5f). Further, analysis of chimerism of CD11b^−^F4/80^+^ thymic macrophages following parabiosis indicated that circulating short-lived adult monocytes likely do not contribute to the thymic macrophage population (Fig. 5g). However, due to the duration of the parabiosis experiment (4 weeks), our results rule out short lived-circulating monocytes as potential parent cells for NR4A1-dependent thymic macrophages but not longer-lived circulating monocytes or progenitors. Utilizing a *Flt3*-cre; *Rosa26*-YFP fate-mapping system, whereby HSC-derived cells are labeled with YFP, we found that the thymic CD11b^−^F4/80^+^ cells either have at some point, or currently express, Flt3 (Fig. 5b). Our bone marrow transplant studies also support the notion that these cells are HSC-derived.

At steady state, thousands of cells die by apoptosis and are replaced every second^28^. These apoptotic bodies are rapidly and efficiently cleared primarily by tissue macrophages, however, a number of both hematopoietic and non-hematopoietic cells also contribute to their clearance^29^. In this report we show that *Nr4a1*-deficient mice fail to effectively clear apoptotic thymocytes *in vivo* following injection with dexamethasone (Fig 6c,d). However, only slight differences in Annexin V^+^7AAD^+^ cells in the thymus were observed between wild type and *Nr4a1*-knockout mice in the absence of Dex at steady state. This is likely due to the presence of other phagocytic cells within the thymus, particularly eosinophils, dendritic cells, and other thymic macrophage subsets that are capable of efferocytosis^30^,^31^. Furthermore, NR4A1 functions in pro-apoptotic pathways in developing thymocytes, and *Nr4a1*-deficient mice display a reduction in thymocyte apoptosis during T-cell selection^32^. Thus the increase in thymocyte survival at steady state in *Nr4a1*-deficient mice may mask an apoptotic cell clearance defect resulting from aberrant thymic macrophage development^32^.

Interestingly, maintenance of thymic homeostasis is altered in *Nr4a1*-deficient mice as these animals display accelerated thymic demise, with a concomitant increase in pro-inflammatory cytokine production (Fig. 7a,b). Further, increased anti-nuclear antibody production is observed in *Nr4a1*^−/−^ mice (Fig. 7c), suggesting that *Nr4a1*-dependent macrophages may be critical mediators of thymic homeostasis and the control of self-tolerance. While these data are likely the result of impaired thymic macrophage development, we cannot formally exclude the possibility that *Nr4a1*-deficiency in other thymic cells also contributes both to the observed accelerated thymic demise and increase in anti-nuclear antibody production. We did not, however, observe evidence of pathologic autoimmunity in these animals at one year of age (data not shown), suggesting that additional factors compensate for the loss of Nr4a1 to prevent autoimmunity.

In sum, we identify a novel subset of NR4A1-dependent CD11b^−^F4/80^+^ macrophages in the thymus. These cells are proliferative, and function to engulf apoptotic thymocytes and maintain tissue homeostasis. These findings highlight the importance of the transcription factor NR4A1 in myeloid cell development, and provide a novel mechanism for tissue and subset specific macrophage development.

## Additional Information

**How to cite this article**: Tacke, R. *et al.* The transcription factor NR4A1 is essential for the development of a novel macrophage subset in the thymus. *Sci. Rep.* doi: 10.1038/srep10055 (2015).

## Supplementary Material

Supplementary Information
