# Supplementary material for: The transcription factor NR4A1 is essential for the development of a novel macrophage subset in the thymus
Source: Sci Rep. 2015 Jun 19;5:10055. doi: 10.1038/srep10055 (PMC4473761; doi:10.1038/srep10055)
Supplement: Supplementary Information [file srep10055-s1.pdf]

Supplementary Figures and Tables

The transcription factor NR4A1 is essential for the development of a novel macrophage subset in the thymus.

Robert Tacke[1], Ingo Hilgendorf[3], Hannah Garner[2], Claire Waterborg[1], Kiwon Park[1], Heba Nowyhed[1], Richard N. Hanna[1], Runpei Wu[1], Filip K Swirski[3], Frederic Geissmann[2], and Catherine C. Hedrick[1]

1) Division of Inflammation Biology, La Jolla Institute for Allergy and Immunology, La Jolla, California, USA.
2) Centre for Molecular and Cellular Biology of Inflammation, King's College London, London, UK.
3) Center for Systems Biology, Massachusetts General Hospital and Harvard Medical School, Boston, Massachusetts, USA.

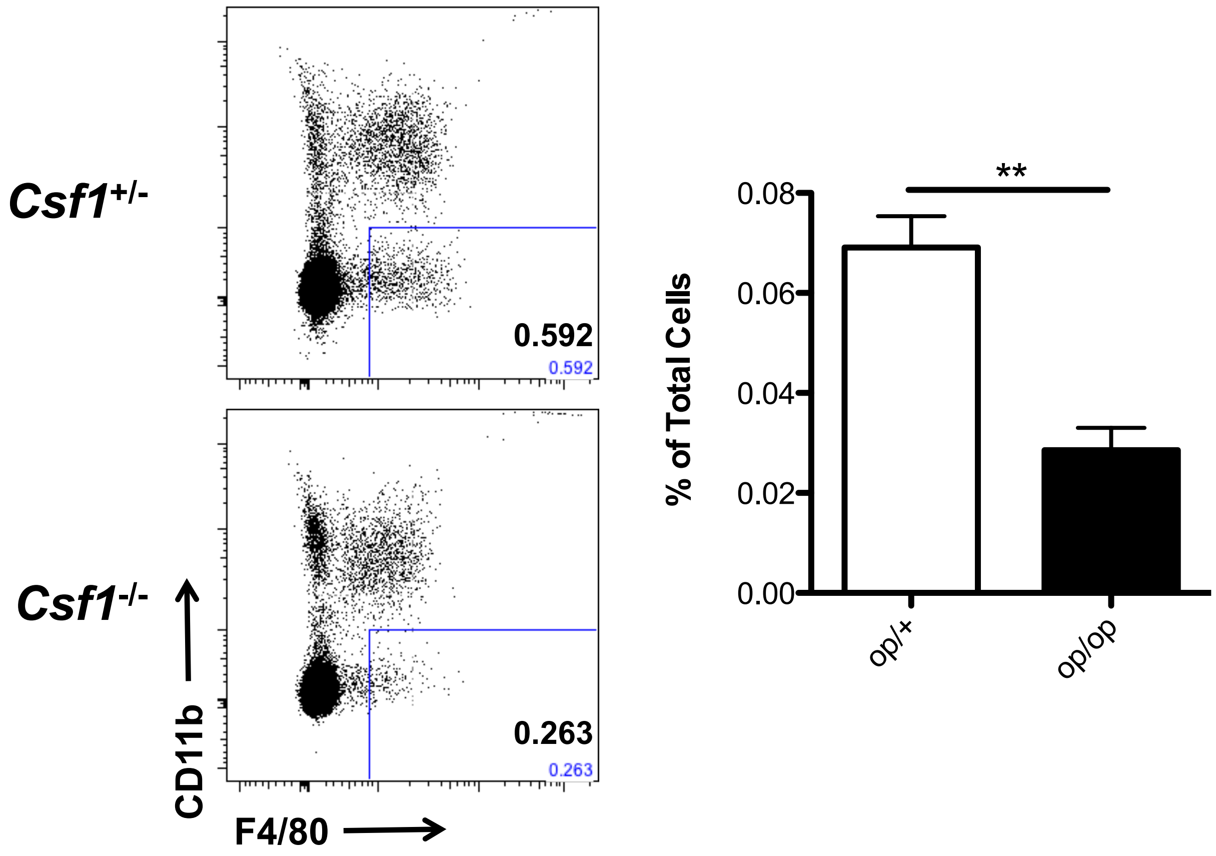

Supplemental Figure 1. Thymic CD11b$^-$F4/80$^+$ cells are CSF1-dependent. Thymus cells from *Csf1*$^{-/+}$(*op/+*) and *Csf1*$^{-/-}$ (*op/op*) mice were stained with antibodies against CD11b and F4/80 and analyzed by flow cytometry. Data representative of two independent experiments. n=6 mice ** $P < 0.01$

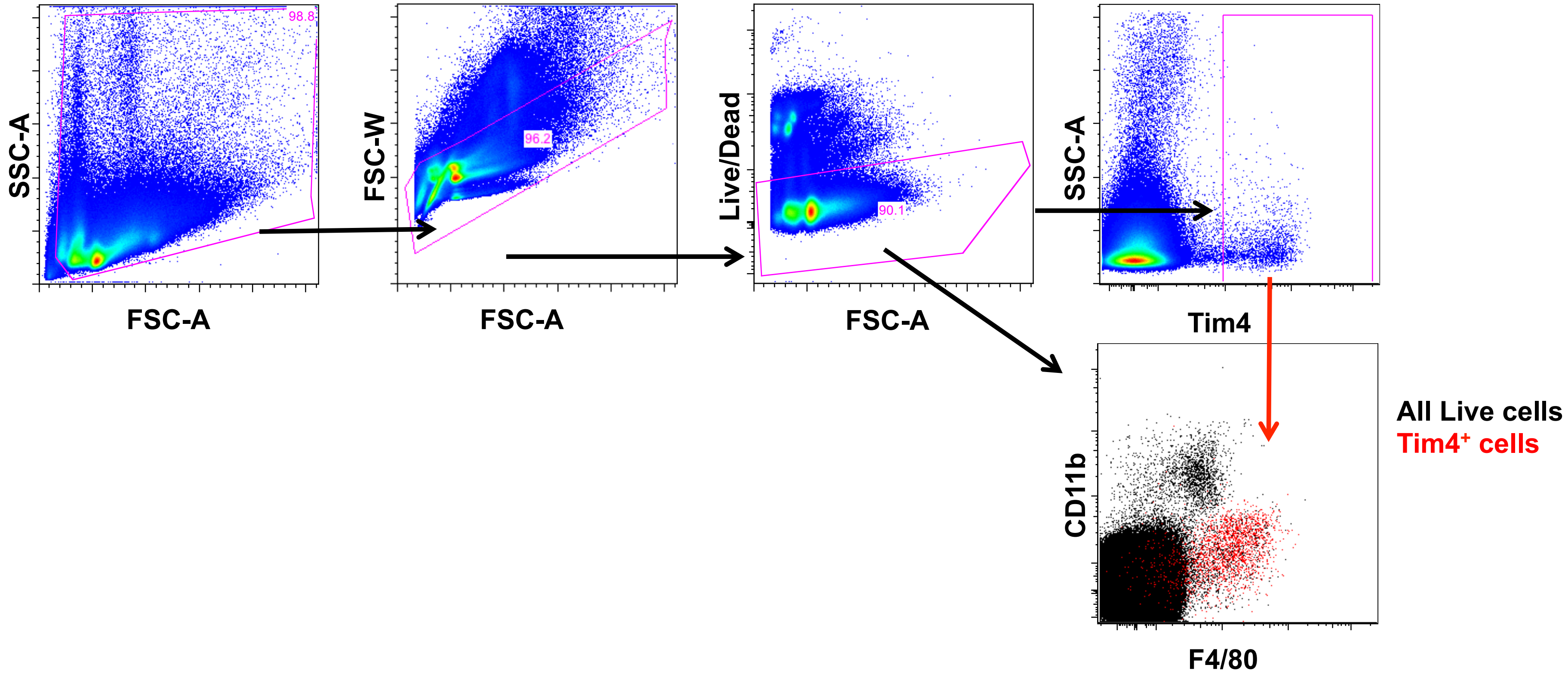

Supplemental Figure 2. Thymic Tim4^{+} cells are CD11b^{-}F4/80^{+} macrophages. Thymus cells from C57BL/6 mice were stained with the indicated antibodies and analyzed by flow cytometry. Tim4^{+} cells(red) were plotted with all live cells(black).

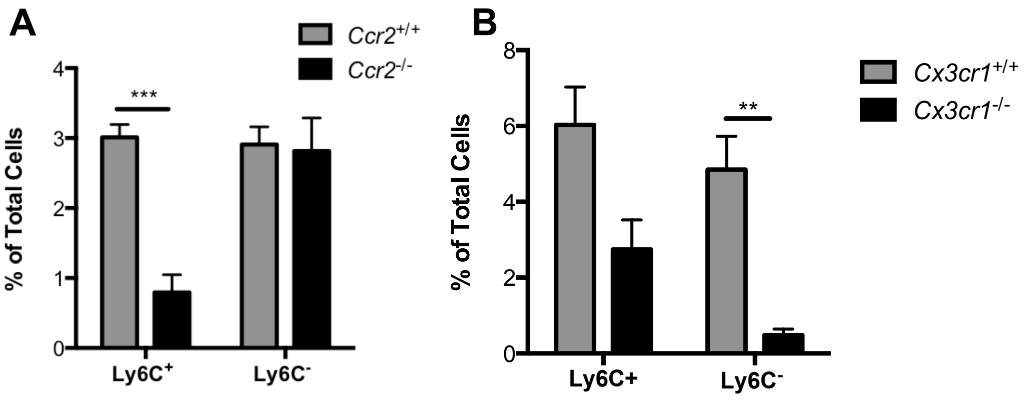

Supplemental Figure 3. Blood monocyte frequencies in *Ccr2$^{-/-}$* and *Cx3cr1$^{-/-}$ mice.* (a and b) Flow cytometric analysis of Ly6C^{+} and Ly6C^{-} monocytes (identified as Lin^{-}CD11b^{+}CD115^{+}Ly6C$^{+/-}$) from the blood of *Ccr2$^{-/-}$ or Cx3cr1$^{-/-}$* mice. Frequency calculated as a percent of the total, live, singlet cells in the blood. Data are representative of at least two independent experiments (n=6 mice). ** $P < 0.01$ *** $P < 0.001$

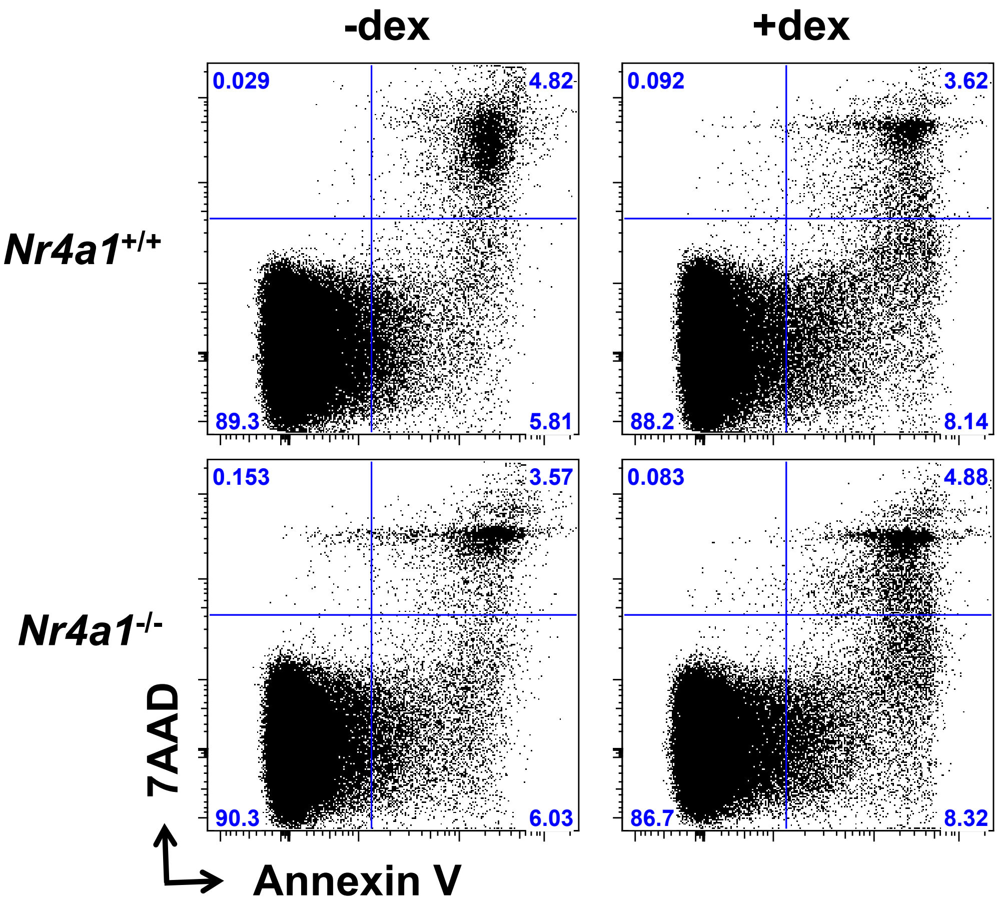

Supplemental Figure 4. No change in induction of thymocyte apoptosis by dexamethasone in *Nr4a1*$^{-/-}$ mice. 7AAD and Annexin V staining from *Nr4a1*$^{+/+}$ and *Nr4a1*$^{-/-}$ mice following 2hr dexamethasone (dex) treatment. Data are representative of at least two independent experiments (n=6 mice)

Antibody	Company	Clone	Secondary (if not directly conjugated)
CD11b	eBioscience	M1/70	
F4/80	eBioscience	BM8	
CD45	BioLegend	30-F11	
Mertk	R&D Systems	BAF591	streptavidin-PE-Cy7 (405206) BioLegend
CD64	BD biosciences	X54-517.1	
Tim4	eBioscience	RMT4-54	
MHCII	eBioscience		M5/114.15.2
SiglecF	BD biosciences	E50-2440	
Ly6G	BioLegend	IA8	
Ly6C	BioLegend	HK1.4	
CD11c	BioLegend	N418	
B220	BioLegend	RA3-6B2	
CD49b	eBioscience	DX5	
Ki67	eBioscience	SolA15	

Supplemental table 1. Antibodies used in this study

Gene	Assay ID
GAPDH	4352932-0912031
Mertk	Mm00434920_m1
Tyro3	Mm00444547_m1
IL6	Mm00446190_m1
IL10	Mm00439614_m1
IL23	Mm01160011_g1
TNF	Mm00443258_m1

Supplemental table 2. Taqman primers used in this study

Gene	Primer Sequence F	Primer Sequence R
AXL	TACCCACCCCATCGTCTGACAGCAC	GGACCCCTGGCCTGAGTGGCATCTA
CD68	AACGCCTTCCGCTGCCC	CGATGCCCGCTGACTCC
MFGE8	CGGGCCAAGACAATGACATC	TCTCTCAGTCTCATTGCACACAAG
STAB2	CTTTGGCAAGCAGCAGCCTG	GTTCTCCAGCTCCCGTTCTC

Supplemental table 3. SYBR green primers used in this study
